# Roles of microRNAs in prenatal chondrogenesis, postnatal chondrogenesis and cartilage-related diseases

**DOI:** 10.1111/jcmm.12161

**Published:** 2013-10-31

**Authors:** Jin Shang, Huan Liu, Yue Zhou

**Affiliations:** Department of Orthopedics, Xinqiao Hospital, Third Military Medical UniversityChongqing, China

**Keywords:** microRNA, chondrogenesis, stem cell, embryonic development, cartilage-related disease

## Abstract

Cartilage has limited repair and regeneration capacity, thus damage of cartilage often results in its dysfunction and even chronic diseases like osteoarthritis (OA). Chondrogenesis induced by tissue-engineering methods is essential to treating cartilage-related diseases. MicroRNAs (miRNAs) are a class of small non-coding single-stranded RNAs which exert their biological effects by binding to the target messenger RNAs (mRNAs), resulting in decay or translation suppression of target mRNAs. There are emerging evidence indicating that miRNAs may play important roles in regulating both prenatal and postnatal chondrogenesis. During embryonic skeletal development, prenatal chondrogenesis is thought to be a precondition for formation of cartilage in developing limbs. Plenty of studies on different types of stem cells have undoubtedly proven their capacity of differentiating into chondrocytes. MiRNAs are found to comprehensively modulate these processes by establishing an interaction network with target genes, transcription factors and cytokines *et al*. In addition, translational application of miRNA technology has also been explored. In this review, we focus on the up-dated progress on regulatory mechanisms of miRNAs in prenatal and postnatal chondrogenesis. In addition, several miRNA target genes and roles of miRNAs in cartilage-related diseases are also discussed. This will contribute to studies of chondrogenesis mechanisms and development of new treating methods.

- Introduction
- MicroRNAs in prenatal chondrogenesis
  –MicroRNAs are essential in prenatal chondrogenesis
  –Expression patterns of miRNAs in prenatal chondrogenesis
  –Functions of miRNAs in prenatal chondrogenesis
- MicroRNAs in postnatal chondrogenesis of stem cells in vitro
- MicroRNAs in chondrogenic differentiation of mesenchymal stem cells
  –Expression patterns of miRNAs during MSC chondrogenic differentiation
  –Functions of miRNAs during MSC chondrogenic differentiation
- MicroRNAs in chondrogenic differentiation of other types of stem cells
- Target genes of miRNAs in chondrogenesis
- MicroRNAs in cartilage-related diseases
  –MicroRNAs in OA
  –MicroRNAs in RA
  –Application of microRNAs in translational medicine
- Conclusion and Prospects

## Introduction

Providing the characteristics of skeletal systems, cartilage is an avascular tissue without nerves, and it consists of only one cell type—chondrocytes. Thus, due to the simplicity of its formation and construction, the repair and regeneration capacity of cartilage is consequently limited, resulting in its vulnerability to changeable immediate environment. In some cases, damage of cartilage resulted from traumatic injuries or autoimmune reactions leads to its dysfunction, and even severe joint diseases, such as osteoarthritis (OA) and rheumatoid arthritis (RA). In recent years, there has been increasing demand for inducing chondrogenesis and regenerating completely functional cartilage to treat cartilage-related diseases 1–2. A number of tissue-engineering methods including cell therapy, gene therapy and biomaterial have been adopted to induce chondrogenesis and obtain functionally improved cartilage 3. However, it is still a challenge to regenerate completely effective and functional cartilage 4. Many metabolic and regulatory problems related with chondrogenesis and cartilage formation are still unknown. Therefore, the detailed mechanisms of chondrogenic differentiation need to be elucidated.

MicroRNAs (miRNAs) are a crowd of small non-coding single-stranded RNAs whose lengths are about 17–24 nucleotides. They often reside outside genes, or sometimes, within introns of other genes. Usually, miRNAs bind to the 3′-untranslated region (3′-UTR) of the target messenger RNAs (mRNAs) through perfect or imperfect complementary base pairing to induce mRNAs’ decay or translational suppression 5. The synthesis of miRNAs in cells is precisely regulated. Firstly, a miRNA gene is transcribed by RNA polymerase II or III in the nucleus, and the transcriptive product is called pri-miRNA, which contains the sequence of the mature miRNA in a stem-loop structure. Then, a pri-miRNA is regularly transferred to be cleaved by a protein complex comprising RNA endonuclease III (known as Dosha) and DGCR8 (DiGeorge syndrome critical region gene 8). This cleaving produces the precursor miRNA (pre-miRNA), which has a short stem-loop of 70–100 nucleotides in the nucleus 6. The pre-miRNA is exported by exportin-5 to the cytoplasm, in which the short stem-loop is removed by Dicer, another RNA endonuclease III, leaving a miRNA duplex alone 7. The double strands then are incorporated into the RNA-induced silencing complex (RISC), where the duplex binds to proteins of the Argonaute family to degrade one strand—passenger miRNA and release the other strand—mature miRNA. The released mature miRNA with RISC contains the seed sequence which interacts with 3′-UTR, or in unusual cases, 5′-UTR and protein coding regions of the target mRNA through fully or partially complementary base pairing 8. When the base pair match is perfect, RISC degrades the target mRNA; otherwise, RISC inhibits the translation of the target mRNA or deadenylates and/or dacaps the target mRNA 9. Up till now, published data have identified more than 1000 miRNAs in human, and it is estimated that they may target about 60% of total human mRNAs 10. Since miRNAs were first discovered in C. elegans in 1993 11–12, a lot of studies have proven that miRNAs exert their functions in a great variety of biological processes including embryo development, cell proliferation, cell differentiation, signal transduction, immune response and apoptosis 13,14.

For many years, the urgent demand of setting up interdisciplinary tissue-engineering strategies to treat cartilage-related diseases has always been addressed by both patients and doctors. Much evidence has proven that miRNAs play important roles in regulating chondrogenesis and pathogenesis of cartilage-related diseases 16. MiRNAs may be developed as new candidates for diagnostic markers and therapeutic targets in treating cartilage-related diseases. So in this review, we focus on miRNA functions in regulating prenatal chondrogenesis of embryonic development and postnatal chondrogenesis of different types of stem cells *in vitro*. In addition, we also discussed several miRNA target genes and roles of miRNAs in cartilage-related diseases.

## MicroRNAs in prenatal chondrogenesis

### MicroRNAs are essential in prenatal chondrogenesis

Chondrogenesis is believed to be the onset of embryonic skeletal development and a premise for formation of cartilage in developing limbs. During this process, mesenchymal cells are recruited and undergo a succession of alterations, including proliferation, progenitor condensation, differentiation, hypertrophy, mineralization, apoptosis and displacement by bone, resulting in cartilage formation and endochondral ossification 17–18. Many factors participate in the regulatory mechanisms of prenatal chondrogenesis, such as transcription factors, growth factors, extracellular matrix (ECM) and miRNAs. The depletion of RNA endonuclease Dicer severely injured proliferating chondrocytes and accelerated post-mitotic chondrocytic hypertrophy, leading to skeletal growth defects, and even deaths of mice 19. This result indicated that miRNAs were essential to chondrocyte survival and inhibition of premature hypertrophic differentiation of chondrocytes in skeletal development.

### Expression patterns of miRNAs in prenatal chondrogenesis

The expression patterns of miRNAs in prenatal chondrogenesis are both species-specific and tissue-specific. In axolotls, two different microarray platforms identified overexpression of miR-21 in mid-bud blastemas compared with stump tissue 20. During chick development, miR-455-3p was expressed in developing long bones 21. In terms of tissue-specific expression patterns of miRNAs, a miRNA expression profiling study of medaka embryos found specific expression of miR-140 and miR-199a in developing cartilage compared with other tissues 19. In the developing zebrafish embryos, 10 miRNAs, including miR-23a, miR-27a, miR-27b, miR-140, miR-140*, miR-145, miR-146, miR-199a, miR-199a* and miR-214, were mainly found in pharyngeal arches, indicating that these miRNAs were highly specific in cartilaginous tissues 22. Moreover, the Solexa sequencing identified both increased and decreased miRNAs only in developing articular cartilage of rats 23. The aforementioned results displayed the species-specific and tissue-specific characteristics of miRNA expression and may provide research targets in the developmental biology.

### Functions of miRNAs in prenatal chondrogenesis

MicroRNAs are critical regulators in prenatal chondrogenesis. In prenatal chondrogenesis, the morphological transition of mesenchymal cells from a fibroblastoid shape to a round one is a persuasive indicator of differentiation 24. In the chick embryonic development, the inhibition of miR-34a led to the rearrangement of actin cytoskeleton by modulating cross-talk between *RhoA* and *Rac1*. At the same time, miR-34a overexpression could cause intensified stress fibres 25. In addition, miR-34a could strongly constrain chondroblasts from migrating by targeting *EphA5* and assist in Interleukin-1β-induced damage of chondrocytes. These findings suggested multiple functions of miR-34a in chick prenatal chondrogenesis 26. Position-dependent chondrogenesis is a common phenomenon in limb development 27. Kim *et al*. showed that by enhancing *Adam9* transcription, miR-142-3p inhibited chondrocyte migration and pre-cartilage condensation and increased cell death. These alterations contributed to the limb type-specific outcome of transforming growth factor-β3 (TGF-β3) upon chick leg *versus* wing mesenchymal cells 28. Similarly, miR-375 was also found to negatively regulate chick chondrocyte migration and pre-cartilage condensation by down-regulating *cadherin-7*^29^. Moreover, Kim *et al*. also confirmed that blockade of miR-221 obviously promoted proliferation of chick chondrogenic progenitors and repression of target *Mdm2* by inhibiting ubiquitylation and proteosomal degradation of Slug protein 30. In terms of control of miRNAs, miR-140 was modulated by transcription factor *Sox9* in the developing zebrafish, however, its function was independent of Sox9b 31. These research findings described above persuasively show that miRNAs play multifunctional and comprehensive roles in prenatal chondrogenesis of mammalian animals.

## MicroRNAs in postnatal chondrogenesis of stem cells *in vitro*

The postnatal chondrogenic differentiation of different types of stem cells *in vitro* has been comprehensively studied and it is under a complex regulatory regime. The important roles of miRNAs in various types of stem cells, such as mesenchymal stem cells (MSCs), adipose-derived stem cells (ADSCs) and unrestricted somatic stem cells (USSCs), have been recognized and discussed. Up to now, a number of significant findings have presented individuals with a clearer picture of miRNA integral part of regulatory mechanisms in chondrogenic differentiation of stem cells.

## MicroRNAs in chondrogenic differentiation of MSCs

Mesenchymal stem cells, also referred to as mesenchymal stromal cells, have the capacity of self-renew and differentiation into multiple cell lineages including osteocytes, chondrocytes, adipocytes, whereas the differentiation capacity into other cell types remains to be controversial 32,33. Many studies have identified miRNA expression profiling during chondrogenic differentiation of MSCs and these findings provide new insights into the detailed mechanisms of MSC chondrogenic differentiation.

### Expression patterns of miRNAs during MSC chondrogenic differentiation

The aberrant expression patterns of miRNAs are identified during MSC chondrogenic differentiation. By using miRNA microarrays, five miRNAs (miR-26b, miR-28, miR-130b, miR-152, miR-193b) were significantly overexpressed in chondrogenic differentiated human MSCs compared with undifferentiated MSCs in two samples 35. Similarly, miRNA microarray analysis identified 10 up-regulated miRNAs (miR-15b, miR-16, miR-23b, miR-27b, miR-140, miR-148, miR-197, miR-222, miR-328 and miR-505) through comparison between human MSCs and human articular chondrocytes 36. Furthermore, in isolated human CD146^+^ MSCs, 12 miRNAs were up-regulated and 24 miRNAs were down-regulated during chondrogenic differentiation 37. In terms of mouse MSCs, eight miRNAs were strongly overexpressed and five miRNAs were under-expressed during TGF-β3-induced chondrogenic differentiation 38. In addition, the expression levels of seven miRNAs altered more than fivefold during chondrogenic differentiation of mouse MSCs 39. It should be noted that different studies found more or less different changes of miRNA expression patterns during chondrogenic differentiation of human MSCs, and so was the case in mouse MSCs. This inconsistency in different findings may be attributed to the various sources of MSCs 40–41, diverse culturing methods and different micro-environments of MSCs.

### Functions of miRNAs during MSC chondrogenic differentiation

The special expression patterns of miRNAs during MSC chondrogenic differentiation are essential to miRNA function. Laine *et al*. found that during chondrogenic differentiation of human MSCs, miR-124 down-regulated *ACAN* expression, whereas miR-199a up-regulated *ACAN* expression 42. ACAN is a marker gene of chondrogenic differentiation. Besides, miR-455-3p acted on TGF-β signalling by targeting *ACVR2B*, *SMAD2* and *CHRDL1* 21. In addition, miR-449a inhibited *Lymphoid Enhancer Binding Factor-1*(*LEF-1*) expression in a dose-dependent and sequence-specific manner during chondrogenesis of human MSCs. And inhibition of *LEF-1* by miR-449a repressed expression of *SOX9*, leading to delayed progression of chondrogenesis 43. Moreover, a small molecule H-89 could induce miR-23b expression, and miR-23b was a positive regulator which therefore promoted chondrogenic differentiation of human MSCs by inhibiting protein kinase A (PKA) signalling 44. During chondrogenic differentiation of mouse MSCs, miR-145 repressed *Sox9* expression and down-regulated mRNA levels such as *Col2a1*, *Comp*, *Acan*, *Col9a2*, which were all chondrogenic marker genes 45. In addition, through modulating transcription factor Smad1, miR-199a* decreased expression of mouse early chondrogenic marker genes 46. These findings clearly showed that by targeting certain genes and transcription factors, miRNAs contributed a lot to the regulation and modulation of chondrogenic differentiation of MSCs.

## MicroRNAs in chondrogenic differentiation of other types of stem cells

Although MSCs show a variety of favourable cellular capacities in tissue engineering, there are other types of stem cells which have their own advantages and could also be good candidates. One example comes from the unrestricted somatic stem cell (hUSSC). USSCs can also differentiate into many cell lineages. Compared with MSCs, USSCs are less mature and have a longer life span and extended telomere length 47–48. By using miRNA microarray and RT-qPCR, Bakhshandeh *et al*. identified many up-regulated and down-regulated miRNAs and their families during USSC chondrogenic differentiation, such as miR-376, miR-624, miR-630 and miR-1268. Particularly, expression of miR-16 decreased about threefold in chondrocytes compared to hUSSCs. However, expression of miR-16 was increased in primary chondrocytes compared with hMSCs 36. The conflicting results for miR-16 may be due to the different characteristic of hUSSCs and hMSCs. Human MSC populations are quite heterogenous. Another reason may be that miR-16 is modulated by some other factors except for chondrogenesis 49. Another example is human adipose-derived stem cell (hADSC). ADSCs are capable of self-renewing and differentiating into multiple cell lineages. Besides, ADSCs are easily available since adipose tissue is abundant 50–51. MiRNA microarray analysis confirmed 12 differentially expressed miRNAs during chondrogenic differentiation of ADSCs, including miR-193b, miR-199a-3p/miR-199b-3p, miR-455-3p, miR-210, miR-381, miR-92a, miR-320c, miR-136, miR-490-5p, miR-4287, miR-BART8* and miR-US25-1* (Table 1). The predicted target genes of these microRNAs were also analysed, which exerted huge effects on chondrogenic differentiation of ADSCs 52. In addition, miR-194 inhibited chondrogenic differentiation of ADSCs by targeting *SOX5* 53. The functions of major miRNAs in chondrogenesis are listed in Table 2. These findings are significant and instructive, but there are still many unknown details which remain to be elucidated in the mechanisms of miRNA function.

**Table 1:** Changes of microRNA expression during chondrogenic differentiation of different stem cells

	Name	Cell types	References
Up-regulated microRNAs	miR-26b	hMSCs	35
	miR-28	hMSCs	35
	miR-130b	hMSCs	35
	miR-152	hMSCs	35
	miR-193b	hMSCs	35
	miR-15b	hMSCs	36
	miR-16	hMSCs	36
	miR-23b	hMSCs	36
	miR-27b	hMSCs	36
	miR-140	hMSCs	36
	miR-148	hMSCs	36
	miR-197	hMSCs	36
	miR-222	hMSCs	36
	miR-328	hMSCs	36
	miR-505	hMSCs	36
	miR-127	Mouse MSCs	38
	miR-140	Mouse MSCs	38
	miR-125b^*^	Mouse MSCs	38
	miR-99a	Mouse MSCs	38
	miR-140^*^	Mouse MSCs	38
	miR-181a-1	Mouse MSCs	38
	let-7f	Mouse MSCs	38
	miR-30a	Mouse MSCs	38
	miR-24	Mouse MSCs	39
	miR-101	Mouse MSCs	39
	miR-124a	Mouse MSCs	39
	miR-199a	Mouse MSCs	39
	miR-199b	Mouse MSCs	39
	miR-1207-5p	hUSSCs	49
	miR-1225-5p	hUSSCs	49
	miR-1246	hUSSCs	49
	miR-1268	hUSSCs	49
	miR-1275	hUSSCs	49
	miR-1287	hUSSCs	49
	miR-1290	hUSSCs	49
	miR-146a	hUSSCs	49
	miR-181a-1	hUSSCs	49
	miR-188-5p	hUSSCs	49
	miR-1915	hUSSCs	49
	miR-210	hUSSCs	49
	miR-630	hUSSCs	49
	miR-638	hUSSCs	49
	miR-193b	hADSCs	52
	miR-199a-3p/miR-199b-3p	hADSCs	52
	miR-455-3p	hADSCs	52
	miR-210	hADSCs	52
	miR-381	hADSCs	52
	miR-92a	hADSCs	52
	miR-320c	hADSCs	52
	miR-136	hADSCs	52
Down-regulated microRNAs	miR-145	Mouse MSCs	38
	miR-212	Mouse MSCs	38
	miR-132	Mouse MSCs	38
	miR-143	Mouse MSCs	38
	miR-125b-3p	Mouse MSCs	38
	miR-18	Mouse MSCs	38
	miR-96	Mouse MSCs	38
	miR-100	hUSSCs	49
	miR-10b	hUSSCs	49
	miR-127-3p	hUSSCs	49
	miR-130a	hUSSCs	49
	miR-143	hUSSCs	49
	miR-145	hUSSCs	49
	miR-16	hUSSCs	49
	miR-221	hUSSCs	49
	miR-376a	hUSSCs	49
	miR-376c	hUSSCs	49
	miR-377	hUSSCs	49
	miR-379	hUSSCs	49
	miR-490-5p	hUSSCs	49
	miR-490-5p	hADSCs	52
	miR-4287	hADSCs	52
	miR-BART8^*^	hADSCs	52
	miR-US25-1^*^	hADSCs	52

**Table 2:** Functions of major microRNA in chondrogenesis

Name	Types of chondrogenesis	Targets	Function	Reference
miR-34a	Prenatal	*RhoA/Rac1*	Rearrange actin cytoskeleton	25
		*EphA5*	Inhibit chondroblast migration	26
miR-142-3p	Prenatal	*Adam9*	Limb type-specific chondrogenesis	28
miR-375	Prenatal	*Cadherin-7*	Inhibit pre-cartilage condensation	29
miR-221	Prenatal	*Mdm2*	Promote progenitor proliferation	30
miR-455-3p	Prenatal	*ACVR2B, SMAD2, CHRDL1*	Affect TGFβ Signalling	21
miR-449a	Postnatal	*LEF-1*	Delayed Chondrogenesis	43
miR-23b	Postnatal	PKA signalling	Promote chondrogenic differentiation	44
miR-145	Postnatal	*Sox9*	Affect chondrogenic differentiation	45
miR-199a^*^	Postnatal	*Smad1*	Decrease chondrogenic marker genes	46
miR-194	Postnatal	*Sox5*	Inhibit chondrogenic differentiation	53

## Target genes of miRNAs in chondrogenesis

Regulating certain target genes is one of the most important ways for miRNAs to exert their multiple functions in chondrogenesis. One miRNA may have many target genes, and one gene may be regulated by many miRNAs. The genes targeted by miRNAs often play vital roles in the overall progression of chondrogenesis.

One of the target genes is *C/EBPβ*. *C/EBPβ* was regulated by several miRNAs during chondrogenic differentiation of hADSCs and its expression level was up-regulated after chondrogenesis 52. In addition, *C/EBPβ* was confirmed to facilitate transition of chondrocytes from proliferative state to hypertrophic state and down-regulation of *C/EBPβ* inhibited progression of OA 54. Moreover, *C/EBPβ* could degrade type II collagen and aggrecan and induce expression of pro-inflammatory cytokines and chemokines 55–56. Besides, by usage of alternative translation, the multi-isoforms of *C/EBPβ* could be obtained from a single mRNA 55,57.

Sox gene family includes over 30 members and plays a master role in chondrogenesis. Smits *et al*. displayed the importance of *Sox5* in chondrogenesis by use of genetically manipulated mice with knockout of *Sox5* 59. Sox5 and Sox6 could be found in differentiating cartilage and they were essential to the control of ECM by enhancing expression of *Col2a1* and *Acan* 60–61. Furthermore, *Sox9* is a strong regulator of chondrogenesis. It induced *Col2a1* expression by activating a 48-bp enhancer residing in the first intron 62 and promoted differentiation of MSCs into chondrocytes.

**Figure 1:**
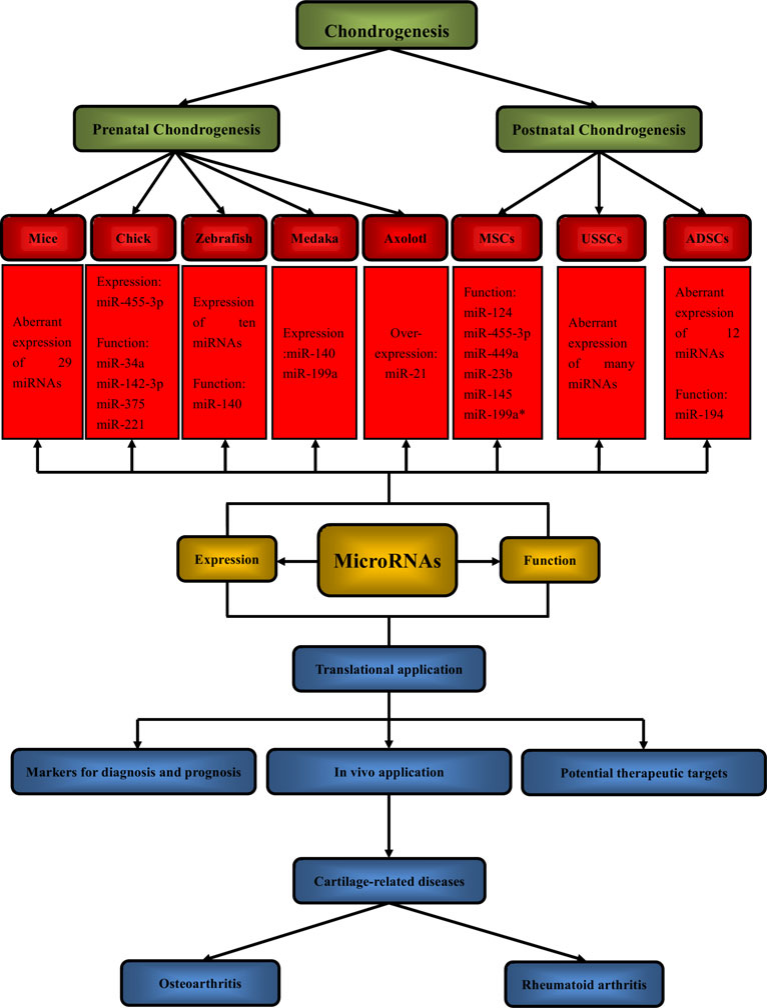
Roles of miRNAs in prenatal chondrogenesis, postnatal chondrogenesis and cartilage-related diseases. Expression and function are two major aspects of miRNAs. During embryonic skeletal development, prenatal chondrogenesis is thought to be a precondition for formation of cartilage in developing limbs. Plenty of studies on different types of adult stem cells have undoubtedly proven their capacity of differentiating into chondrocytes. MiRNAs are found to comprehensively modulate these processes by establishing an interaction network with target genes. In terms of translational application, miRNA technology has great potential to be applied to treat cartilage-related diseases, such as osteoarthritis and rheumatoid arthritis.

In terms of target gene *Adam9*, up-regulation of *Adam9* could lead to apoptosis of chondrogenic progenitors and inhibition of mesenchymal cell migration 28. Besides, Adam9 could bind to myeloma cells with the help of ανβ5 integrin 63 and promote fibroblast adhesion by interacting with α6β1 integrin 64. Moreover, by targeting several integrins, *ADAM9* enhanced invasion of cancer cells 65. The regulation mechanisms of prenatal and postnatal chondrogenesis by miRNAs through targeting genes are presented in Figure 1.

## MicroRNAs in cartilage-related diseases

MicroRNAs are highly involved in cartilage-related diseases. These diseases, such as OA, RA and psoriatic arthritis, often lead to severe joint pain and even disability of elderly persons 66,67. A lot of studies have been done to elucidate the miRNA roles in the pathogenesis and progression of these diseases.

### MicroRNAs in OA

Osteoarthritis is a commonly seen chronic joint disease. The pathogenesis of OA is closely related to the disrupted homeostatic balance between synthesis and degradation of ECM, resulting in cartilage dysfunction and repair failure 69,70.Up till now, effective treatments in OA therapies are limited and the molecular mechanisms underlying the onset and development of OA need to be elucidated urgently. There have been evidences that miRNAs are highly involved in the pathogenesis and progression of OA. Microarray analysis found nine up-regulated miRNAs (miR-16, miR-22, miR-23b, miR-30b, miR-103, miR-223, miR-377, miR-483 and miR-509) and seven down-regulated miRNAs (miR-25, miR-26a, miR-29a, miR-140, miR-210, miR-337 and miR-373) in OA cartilage compared with normal cartilage 72. Inflammatory mediators often participate in OA pathogenesis. Miyaki *et al*. found that the expression of miR-140 was inhibited when chondrocytes were treated with IL-1β, indicating the role of miR-140 in OA pathogenesis 36. Moreover, by generating a mouse line with miR-140 deletion, this group proved that miR-140^−/−^ mice showed age-related OA-like changes. Meanwhile, transgenic mice with overexpressed miR140 showed resistance to joint damage from OA. This study clearly revealed the critical roles of miR-140 in OA 73.

### MicroRNAs in RA

Rheumatoid arthritis is an autoimmune disease which is characterized by chronically inflammatory synovial tissue and irreversible joint damage 67. Recent studies have suggested that miRNAs play crucial roles in RA pathogenesis. Overexpression of miR-16, miR-132, miR-146a and miR-155 was detected in peripheral blood mononuclear cells (PBMCs) from RA patients 74. In addition, the increased expression level of miR-124 was identified in RA synoviocytes 75. MiR-124 was found to play a role in cell proliferation. In *in vivo* experiments, Nakasa *et al*. administered double-stranded miR-146a to prevent joint destruction in arthritic mice 76. This finding indicated the therapeutic potential of miR-146a in treating RA.

### Application of microRNAs in translational medicine

It is crucial to develop methods for *in vivo* application of miRNA technology to regenerate damaged cartilage tissue in clinics. Up till now, intra-articular injection and intravenous injection have showed their great potential. Intra-articular injection of double-stranded miRNA-15a induced cell apoptosis in the synovium of mice with autoantibody-mediated arthritis 77. In addition, administration of miR-210 by intra-articular injection effectively promoted the healing of partly damaged anterior cruciate ligaments (ACLs) 78. Moreover, intravenous injection of miR-146a could protect joint from inflammatory damage in arthritic mice 76. Individuals may learn a lot from these trials and develop new methods for *in vivo* application of miRNA technology. Roles of miRNAs in prenatal chondrogenesis, postnatal chondrogenesis and cartilage-related diseases are presented in Figure 1.

## Conclusion and prospects

Chondrogenesis is critical in cartilage repair and regeneration. A great amount of studies have proven that miRNAs are important multifunctional regulators in chondrogenesis of embryos and stem cells. MiRNAs exert their functions through interactions with their targets, which could be genes, transcription factors, signalling pathways and intracellular functional molecules. Despite the numerous findings about miRNAs, there are still many questions on regulatory mechanisms of miRNAs which remain to be illustrated. The expression levels of miRNAs during chondrogenesis are not static, so information about the continuous variation of miRNA expression levels would help know more about miRNA roles in chondrogenesis. In addition, it should be noted that miRNAs themselves are modulated in various manners. Thus in most cases, miRNAs and their targets often interact with each other to form a whole regulatory network to affect multiple cellular activities. Much more attention should be paid on this network. Besides, several studies found that miRNAs can be packed into microvesicles and secreted outside as exosomes from one cell to another 79–80. This finding indicated extracellular regulation of miRNAs. In terms of disorders, many cartilage-related diseases are accompanied by various pathological processes such as hypoxia, inflammation and stress. So the exact roles of miRNAs in these pathological conditions need further description and elucidation. To sum up, miRNAs are highly involved in the prenatal and postnatal chondrogenesis, and also play important roles in cartilage-related diseases. We are sincerely hopeful that more breakthroughs in regulatory mechanisms of miRNAs will be made, and effective and applicable therapeutic methods can be developed to treat cartilage-related diseases to benefit countless patients.
